# Congenital Erythropoietic Porphyria with Persistent Severe Biochemical Abnormalities and a Non-Mutilating Clinical Course: A Case Report

**DOI:** 10.3390/reports9010065

**Published:** 2026-02-16

**Authors:** Supriya Peshin, Ehab Takrori, Kaneez S. Khan, Bilal Rahimuddin, Sanjaya K. Upadhyaya, Pintu K. Gami, Sakshi Singal

**Affiliations:** 1 Department of Internal Medicine, Norton Community Hospital, Norton, VA 24723, USA; bilal.rahimuddin@balladhealth.org (B.R.); medsanup400@gmail.com (S.K.U.); 2 College of Medicine, Alfaisal University, Riyadh 11533, Saudi Arabia; etakrori@gmail.com; 3 Al-Ameen Medical College, Bijapur 586108, India; seemakhan2786@gmail.com; 4 School of Medical Sciences, Kathmandu University, Dhulikhel 45209, Nepal; meetwithgami@gmail.com; 5 Department of Hematology & Oncology, East Tennessee State University, Johnson City, TN 37614, USA

**Keywords:** congenital erythropoietic porphyria, Günther disease, erythrocyte porphyrins, photosensitivity, afamelanotide, UROS mutations

## Abstract

**Background and Clinical Significance:** Congenital erythropoietic porphyria (CEP), also known as Günther disease, is a rare autosomal recessive porphyria caused by a deficiency of uroporphyrinogen III synthase, leading to the accumulation of phototoxic type I porphyrins. CEP classically presents in infancy with severe photosensitivity, blistering, scarring, and hemolytic anemia; however, significant phenotypic variability has increasingly been recognized. **Case Presentation:** We report a 32-year-old woman diagnosed with CEP in early infancy who demonstrated persistently and profoundly elevated erythrocyte porphyrin levels over more than a decade, yet who followed a relatively non-mutilating clinical course. Genetic testing identified a low-penetrance intronic UROS variant typically associated with erythropoietic protoporphyria, underscoring diagnostic challenges and genotype–phenotype discordance. The patient experienced marked improvement in photosensitivity and burning pain after initiation of afamelanotide, without the need for transfusion therapy or stem cell transplantation. **Conclusions:** This case highlights the heterogeneity of CEP, the importance of long-term biochemical follow up, and the potential role of afamelanotide in improving quality of life for selected patients with CEP.

## 1. Introduction and Clinical Significance

Congenital erythropoietic porphyria (CEP), also known as Günther disease, is a rare autosomal recessive disease of heme biosynthesis caused by deficient activity of uroporphyrinogen III synthase (UROS) [1]. CEP belongs to the group of erythropoietic porphyria and is characterized by the accumulation of non-physiologic type I porphyrins, predominantly uroporphyrin I and coproporphyrin I, within erythroid cells, plasma, skin, and other tissues [1,2,3]. These phototoxic porphyrins lead to severe cutaneous photosensitivity and variable hematologic manifestations, making CEP one of the most clinically debilitating porphyria.

CEP most commonly presents in early infancy with dark or reddish urine, blistering and erosions of sun-exposed skin, hypertrichosis, erythrodontia, and chronic hemolytic anemia [4]. The cutaneous manifestations are driven by porphyrin activation by visible light, particularly wavelengths in the Soret band (400–410 nm), resulting in oxidative tissue injury [5,6]. Repeated phototoxic damage often leads to scarring, deformity, and mutilation of exposed areas such as the hands, face, and ears [1,2,3]. Historically, CEP has been regarded as a severe and relentlessly progressive disease with significant morbidity and reduced quality of life.

Despite this classic description, increasing evidence suggests that CEP exhibits substantial phenotypic heterogeneity. Reports of milder disease, delayed presentation, and even adult-onset CEP challenge the traditional view of CEP as universally devastating [7,8,9]. Adult-onset CEP cases have been described with attenuated cutaneous findings, hematologic abnormalities, or atypical presentations, highlighting the importance of maintaining diagnostic suspicion even outside infancy [8,9]. This variability is attributed to differences in residual UROS activity, genetic modifiers, iron metabolism, and environmental exposures [5,9,10,11,12].

Diagnosis of CEP relies on a combination of clinical features, markedly elevated erythrocyte and urinary porphyrins with a predominance of type I isomers, and molecular confirmation of pathogenic UROS variants [1,3,10]. However, genotype–phenotype correlations are imperfect, and low-penetrance or intronic variants may complicate interpretation, particularly in patients with atypical presentations [9,10]. As a result, biochemical evaluation remains central to diagnosis and disease classification.

Management of CEP is challenging due to its rarity and the absence of standardized treatment guidelines. Traditional management strategies include lifelong photoprotection, wound care, treatment of hemolytic anemia, and supportive management of complications [2,5,12]. In severe cases, transfusion therapy, splenectomy, or hematopoietic stem cell transplantation (HSCT) have been employed, with HSCT representing the only curative option to date [13,14]. However, these interventions carry significant risk and are typically reserved for patients with severe or progressive disease.

Advances in the understanding of erythropoietic porphyria have led to the exploration of therapies initially developed for erythropoietic protoporphyria (EPP), including afamelanotide, a melanocortin analogue that increases eumelanin production and improves tolerance to visible light exposure [4,15]. While approved for EPP, experience with afamelanotide in CEP remains limited, and further studies are needed to better define its efficacy and safety in this population.

Here, we present the case of a woman with CEP diagnosed in early infancy who demonstrated persistently severe biochemical abnormalities over more than a decade, yet who followed a relatively non-mutilating clinical course and experienced symptomatic improvement with afamelanotide. This case highlights the phenotypic variability of CEP, the importance of long-term biochemical monitoring, and the potential applicability of newer therapeutic approaches in selected patients.

This case is reported due to its rarity and unique clinical course, characterized by persistently extreme erythrocyte porphyrin elevations over more than a decade in the absence of progressive mutilation, a documented genotype–phenotype discordance involving a low-penetrance UROS variant, and symptomatic improvement with afamelanotide therapy. Together, these features provide novel insight into the phenotypic heterogeneity and evolving therapeutic considerations in congenital erythropoietic porphyria.

## 2. Case Presentation

The patient is a 32-year-old female with a past medical history significant for congenital erythropoietic porphyria, psoriasis, anxiety/depression, and severe intolerance to intravenous iron, who presented to the Hematology-Oncology clinic for routine follow-up. She was diagnosed with CEP at 2 weeks of age following extensive evaluation, including bone marrow testing. During infancy and childhood, she experienced severe photosensitivity with recurrent second- and third-degree burn injuries following sun exposure, resulting in residual scarring of the dorsal hands and forearms. Early management included beta-carotene supplementation, topical burn therapy, antihistamines, and strict photoprotection.

**Figure 1:**
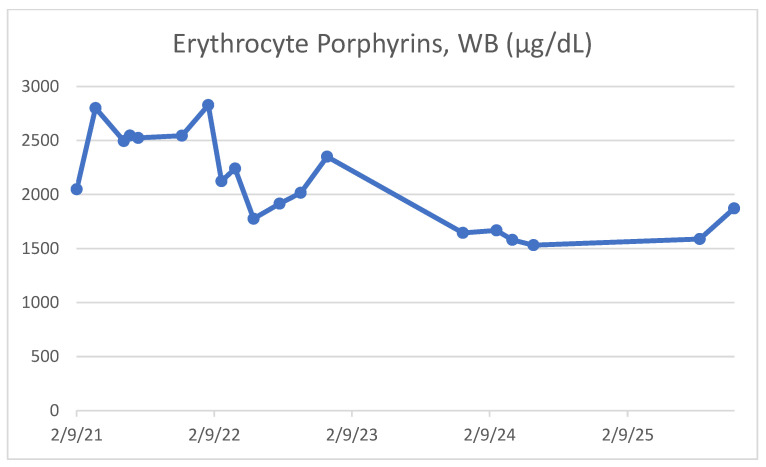
Serial erythrocyte porphyrin measurements over time in a patient with congenital erythropoietic porphyria. Values remained markedly elevated and consistently exceeded the upper limit of normal over more than four years, despite a relatively non-mutilating clinical course and symptomatic improvement following afamelanotide therapy.

Approximately five years prior to presentation, she was referred to a porphyria specialist for further evaluation after markedly elevated erythrocyte porphyrin levels were identified (2048 µg/dL; Figure 1). Erythrocyte porphyrin measurements were performed on whole blood using standardized quantitative fluorometric analysis at a reference laboratory experienced in porphyria diagnostics. Total erythrocyte porphyrins were reported as micrograms per deciliter (µg/dL), with a reference range < 30 µg/dL. Serial measurements consistently demonstrated values exceeding 50-fold the upper limit of normal, with a predominance of type I porphyrins consistent with congenital erythropoietic porphyria. Genetic testing showed a pathogenic low-penetrance intronic UROS variant (c.315-48T>C), which is the most prevalent variant associated with autosomal recessive erythropoietic protoporphyria (EPP). Bone marrow testing was deferred in light of confirmatory genetic and biochemical findings. Bone marrow evaluation was deferred because the diagnosis of congenital erythropoietic porphyria was supported by a long-standing clinical history beginning in infancy, persistently extreme erythrocyte porphyrin elevations with a characteristic biochemical pattern, and molecular identification of a UROS variant. In the absence of progressive cytopenias, worsening hemolysis, or concern for alternative marrow pathology, repeat invasive evaluation was not felt to be clinically indicated. She was subsequently initiated on afamelanotide therapy four years prior to the current visit, with reported improvement in photosensitivity despite persistently elevated erythrocyte porphyrin levels (Table 1). Afamelanotide was administered as a subcutaneous implant at standard dosing intervals consistent with protocols used for erythropoietic protoporphyria, with repeat implantation approximately every two to three months during periods of anticipated sun exposure. The patient was monitored clinically for treatment response and adverse effects at regular follow-up visits. She reported significant improvement in photosensitivity, including reduced burning pain and fewer severe phototoxic reactions, without experiencing treatment-limiting adverse effects. No abnormalities in laboratory monitoring or implant-related complications were observed.

**Figure 2:**
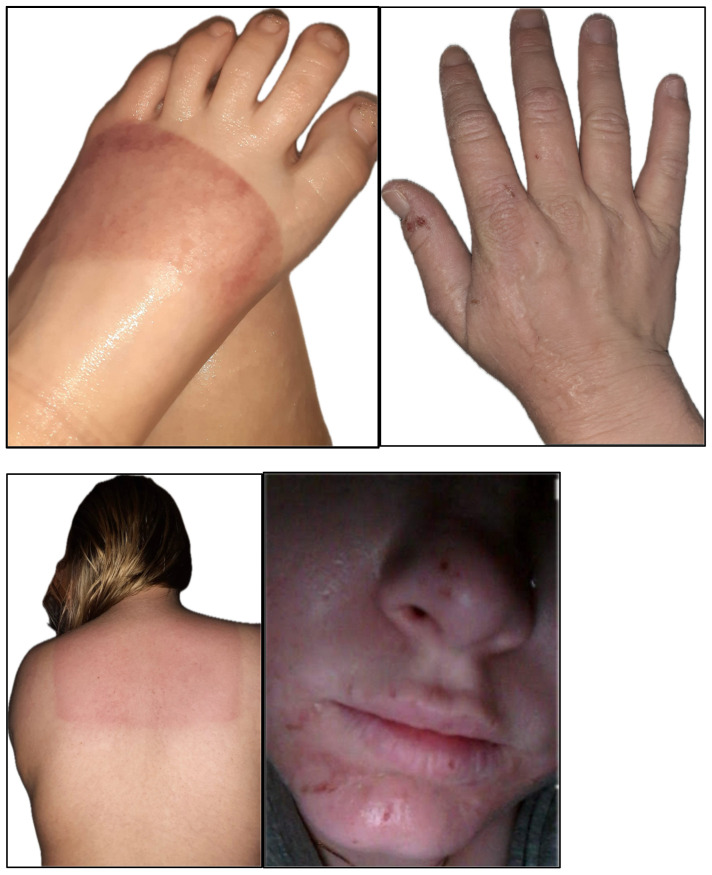
Clinical photographs demonstrating photosensitivity-related cutaneous findings in a patient with congenital erythropoietic porphyria.

On examination, the patient demonstrated cutaneous changes consistent with chronic photosensitivity involving multiple sun-exposed areas. Clinical photographs illustrate sharply demarcated phototoxic injury of the forefoot, scarring of the dorsal hand, patterned sunburn of the upper back corresponding to clothing exposure, and residual facial scarring involving the nose and chin, without evidence of mutilation or tissue loss (Figure 2). The patient remained stable on afamelanotide therapy and continued to practice strict photoprotection, reporting decreased frequency and severity of burn injuries since treatment initiation.

(A) The forefoot shows a sharply demarcated band of sunburn measuring approximately 6–8 cm in width across the dorsal aspect, consistent with phototoxic injury.
(B) The dorsal hand demonstrates residual scarring, most prominent along the thumb, from prior photosensitivity-related injury.
(C) The upper back shows a geometric area of sunburn corresponding to the lower border of clothing, with greater exposure and erythema of the uncovered upper back.
(D) The face demonstrates residual scarring involving the nose and chin with associated cheilitis. No evidence of mutilation, tissue loss, or auto-amputation is observed.

## 3. Discussion

Congenital erythropoietic porphyria (CEP) is an exceptionally rare autosomal recessive porphyria caused by a deficiency of uroporphyrinogen III synthase (UROS), resulting in the accumulation of type I porphyrins and severe photosensitivity. Although classically described as a devastating, mutilating disorder of infancy, CEP is now recognized as a disease with broad phenotypic variability [1]. The present case exemplifies this spectrum: despite a biochemical profile showing persistently extreme erythrocyte porphyrin elevations for more than a decade, the patient’s clinical course has remained non-mutilating, relatively stable, and significantly improved with afamelanotide therapy.

The validity and clinical relevance of this report lie in the combination of long-term biochemical follow-up, atypical genotype-phenotype correlation, and therapeutic response observed in a single patient with CEP. Such integrated longitudinal data are rarely reported and contribute meaningful evidence to the limited literature on non-mutilating CEP phenotypes.

### 3.1. Phenotypic Spectrum of CEP

Classic CEP typically presents during infancy with severe photosensitivity, vesicles, bullae, erosions, and mutilating scarring of sun-exposed areas, including hands, ears, and face [2,3]. Patients often develop erythrodontia, hypertrichosis, splenomegaly, and hemolytic anemia [1]. However, more recent reports describe mild, attenuated, or non-mutilating variants in which patients exhibit lifelong photosensitivity but do not develop progressive disfigurement [7,8,9].

The current patient represents this non-mutilating phenotype, despite decades of markedly elevated erythrocyte porphyrins (>1500–2500 µg/dL). Her early childhood blistering decreased over time, and she did not develop the severe scarring or deformity characteristics of classic CEP. This is consistent with published cases documenting milder disease presentations, sometimes without scarring or hemolysis, and occasionally with later onset [5,7,8]. Such cases underscore that porphyrin levels alone do not predict severity, and that genotype, residual enzyme activity, iron metabolism, and other modifying influences significantly shape disease expression [5,9,10,11,16]. Compared with other reported non-mutilating CEP phenotypes, this case shares the absence of progressive tissue loss or auto-amputation despite lifelong photosensitivity, but it is notable for the persistence of extremely elevated erythrocyte porphyrin levels over more than a decade. Previously described non-mutilating cases have often been associated with lower porphyrin burdens, later onset, or partial biochemical attenuation over time [7,8,9]. In contrast, the sustained severity of biochemical abnormalities in this patient highlights a unique dissociation between porphyrin burden and clinical manifestation, further supporting the concept that additional genetic and environmental modifiers influence disease expression. This case, therefore, expands the spectrum of reported non-mutilating CEP phenotypes by demonstrating long-term clinical stability despite persistently extreme biochemical derangement.

This case is particularly valuable because longitudinal biochemical data extending over a decade are rarely reported. The persistently extreme porphyrin elevation, despite relatively stable clinical manifestations, provides insight into the natural history of non-mutilating CEP.

### 3.2. Molecular and Pathophysiologic Considerations

CEP results from biallelic pathogenic variations in UROS, leading to impaired conversion of hydroxymethylbilane into uroporphyrinogen III. The deficiency causes the spontaneous formation of type I porphyrin isomers, which cannot enter downstream heme synthesis and instead accumulate in tissues where they generate phototoxic injury [1].

Genotype–phenotype correlation in CEP is complex. Some variants, such as the well-studied C73R allele, produce nearly absent UROS activity and aggressive disease [10]. Other mutations allow partial activity, explaining milder phenotypes. Furthermore, modifying factors such as iron status, erythropoietic rate, and additional genetic modifiers influence the severity of cutaneous and hematologic manifestations [9,11,16].

The identification of the c.315-48T>C intronic variant, a low-penetrance allele more commonly associated with erythropoietic protoporphyria (EPP), adds complexity to this case. Although the biochemical pattern clearly indicates CEP rather than EPP, this finding highlights the overlap between erythropoietic porphyria, the imperfect nature of genotype-only diagnosis, and the importance of integrating biochemical, clinical, and molecular findings [9,12]. Such overlap has been observed in other reports demonstrating unexpected genotype-phenotype mismatches, supporting the concept of a continuum of erythropoietic porphyria.

### 3.3. Diagnostic Considerations

Diagnosis of CEP typically relies on:

1. Characteristic clinical findings, particularly blistering and scarring of sun-exposed areas [1,2,3,4].
2. Markedly increased erythrocyte and urine porphyrins, predominantly URO-I and COPRO-I [6,15].
3. Demonstration of reduced UROS activity (not always available) [1].
4. Genetic confirmation of UROS mutations [10].

This patient’s biochemical profile, with persistently extreme erythrocyte porphyrin levels, strongly supports CEP. Erythrocyte porphyrins remain chronically elevated because porphyrins are tightly bound within circulating red cells [6]. This contrasts with EPP, where protoporphyrin levels may fluctuate alongside environmental triggers and erythropoietic activity [12].

One diagnostic challenge illustrated by this case is the potential for misclassification when genetic variants associated with EPP or low-penetrance alleles are detected. The biochemical profile remains the most reliable discriminator in such scenarios [12].

### 3.4. Management Strategies and Therapeutic Lessons

#### 3.4.1. Photoprotection

Photoprotection remains the cornerstone of management for all photosensitive porphyria. Avoidance of visible-light wavelengths around the Soret band (400–410 nm) is essential. Patients often require lifelong environmental modification and protective clothing [5].

#### 3.4.2. Afamelanotide

A significant finding in this case is the patient’s meaningful symptom improvement after initiation of afamelanotide, a melanocortin analogue FDA-approved for EPP. Leaf et al. describe how afamelanotide increases eumelanin density, thereby extending pain-free light exposure time in erythropoietic porphyria [12,15]. Although off-label for CEP, the biologic rationale is applicable because both CEP and EPP involve phototoxic activation of porphyrins by visible light. The positive outcome observed reinforces the potential benefit of afamelanotide as an adjunctive therapy in selected CEP patients, particularly those seeking improved quality of life.

#### 3.4.3. Iron Modulation

Iron influences heme biosynthesis and may modify porphyrin production. Studies suggest that inducing mild iron deficiency can reduce erythropoietic drive and porphyrin accumulation, while excess iron may worsen photosensitivity [9,12]. The patient’s intolerance to IV iron and chronic nausea highlight the importance of individualized iron management.

#### 3.4.4. Transfusion Support and Splenectomy

Transfusions have historically been used to suppress erythropoiesis and reduce porphyrin production in severe CEP [2,3]. Splenectomy was once considered for hemolytic anemia, but it can worsen cutaneous disease in some patients [13].

#### 3.4.5. Hematopoietic Stem Cell Transplantation (HSCT)

HSCT remains the only curative therapy for CEP. Successful transplants show complete normalization of UROS activity, resolution of hemolytic anemia, and dramatic improvement in skin disease [14]. However, due to significant risks, HSCT is reserved for severe, progressive, or mutilating cases, criteria that this patient does not meet.

#### 3.4.6. Emerging Therapies

Newer approaches, including gene therapy, pharmacologic stabilization of mutant UROS proteins, and small-molecule chaperones, show promise in preclinical work [11,12]. These developments reflect a shift toward targeted correction of the underlying metabolic defect.

### 3.5. Systemic Symptoms and Quality of Life Considerations

Patients with erythropoietic porphyria frequently report systemic manifestations during phototoxic episodes, including fatigue, malaise, dehydration, and gastrointestinal distress [15]. These symptoms are believed to result from cytokine release triggered by porphyrin activation. This patient’s chronic nausea, abdominal pain, and fatigue may reflect these mechanisms, compounded by the psychosocial burden of lifelong photoprotection.

## 4. Conclusions

This case is reported due to its rarity and clinical validity, characterized by persistently extreme erythrocyte porphyrin elevations over more than a decade in the absence of progressive mutilation, together with atypical genotype–phenotype correlation and therapeutic response. This patient exemplifies the expanding phenotypic spectrum of CEP, demonstrating that extremely elevated porphyrin levels do not necessarily predict mutilating disease. Her positive therapeutic response to afamelanotide provides valuable evidence supporting broader consideration of melanocortin analogues in CEP management. By combining decades-long biochemical data, atypical genotyping, and detailed clinical evolution, this case strengthens understanding of CEP heterogeneity and highlights opportunities for improved personalized care and future therapeutic innovation.

## Figures and Tables

**Table 1:** Longitudinal erythrocyte porphyrin measurements in a patient with congenital erythropoietic porphyria demonstrate persistently severe biochemical abnormalities over more than four years. Despite sustained porphyrin levels exceeding 50-fold the upper limit of normal, the patient followed a relatively non-mutilating clinical course with symptomatic improvement following afamelanotide therapy.

Date	Erythrocyte Porphyrins, WB (µg/dL; Normal < 30)	Interpretation
9 February 2021	2048	Markedly elevated
31 March 2021	2800	Markedly elevated
14 June 2021	2495	Markedly elevated
30 June 2021	2545	Markedly elevated
22 July 2021	2524	Markedly elevated
15 November 2021	2544	Markedly elevated
24 January 2022	2828	Markedly elevated
28 February 2022	2124	Markedly elevated
5 April 2022	2240	Markedly elevated
24 May 2022	1776	Markedly elevated
1 August 2022	1915	Markedly elevated
26 September 2022	2016	Markedly elevated
5 December 2022	2350	Markedly elevated
30 November 2023	1645	Markedly elevated
27 February 2024	1668	Markedly elevated
9 April 2024	1580	Markedly elevated
4 June 2024	1532	Markedly elevated
19 August 2025	1588	Markedly elevated
18 November 2025	1872	Markedly elevated

## Data Availability

The original contributions presented in this study are included in the article. Further inquiries can be directed to the corresponding author.

## References

[1] Erwin A., Balwani M., Desnick R.J., Adam M.P., Bick S., Mirzaa G.M., Pagon R.A., Wallace S.E., Amemiya A. (2013). Porphyrias Consortium of the NIH-Sponsored Rare Diseases Clinical Research Network. Congenital Erythropoietic Porphyria. GeneReviews^®^ [Internet].

[2] Fritsch C., Bolsen K., Ruzicka T., Goerz G. (1997). Congenital erythropoietic porphyria. J. Am. Acad. Dermatol..

[3] Puy H., Gouya L., Deybach J.C. (2010). Porphyrias. Lancet.

[4] Saikrishna P., Palaniswamy G., Pillikunte Doddareddy N., Ishfaq L., Zargar M.N., Wafa Eranhikkal F., Sahu S. (2024). Congenital Erythropoietic Porphyria: A Rare Inherited Disorder. Cureus.

[5] Murphy A., Gibson G., Elder G.H., Otridge B.A., Murphy G.M. (1995). Adult-onset congenital erythropoietic porphyria (Günther’s disease) presenting with thrombocytopenia. J. R. Soc. Med..

[6] To-Figueras J., Erwin A.L., Aguilera P., Millet O., Desnick R.J. (2024). Congenital erythropoietic porphyria. Liver Int..

[7] Lehmann P., Schwarz T. (2011). Photodermatoses: Diagnosis and treatment. Dtsch. Arztebl. Int..

[8] Desnick R.J., Glass I.A., Xu W., Solis C., Astrin K.H. (1998). Molecular genetics of congenital erythropoietic porphyria. Semin. Liver Dis..

[9] Balwani M., Desnick R.J. (2012). The porphyrias: Advances in diagnosis and treatment. Blood.

[10] Cappellini M.D. (2007). Coagulation in the Pathophysiology of Hemolytic Anemias. Hematol. Am. Soc. Hematol. Educ. Program.

[11] Tintle S., Alikhan A., Horner M.E., Hand J.L., Davis D.M.R. (2014). Cutaneous porphyrias part II: Treatment strategies. Int. J. Dermatol..

[12] Lecha M., Puy H., Deybach J.C. (2009). Erythropoietic protoporphyria. Orphanet J. Rare Dis..

[13] Poh-Fitzpatrick M.B. (1985). Porphyrin-sensitized cutaneous photosensitivity: Pathogenesis and treatment. Clin. Dermatol..

[14] Leaf R.K., Dickey A.K. (2023). How I treat erythropoietic protoporphyria and X-linked protoporphyria. Blood.

[15] Shetty A.K., Ode D., Galen W.K., Warrier R.P. (1998). Successful splenectomy for congenital erythropoietic porphyria (Gunther’s disease). J. Pediatr. Hematol./Oncol..

[16] Besnard C., Schmitt C., Galmiche-Rolland L., Debray D., Fabre M., Molina T., Gouya L., Ged C., Castelle M., Cavazzana M. (2020). Bone Marrow Transplantation in Congenital Erythropoietic Porphyria: Sustained Efficacy but Unexpected Liver Dysfunction. Biol. Blood Marrow Transplant..

